# Association between the insulin-like growth factor 1 gene rs2195239 and rs2162679 polymorphisms and cancer risk: a meta-analysis

**DOI:** 10.1186/s12881-019-0749-3

**Published:** 2019-01-17

**Authors:** Gui-Ping Xu, Wei-Xian Chen, Qing Zhao, Hua Zhou, Shi-Zhi Chen, Li-Fang Wu

**Affiliations:** 1 Transfusion Department, the Second Affiliated Hospital of Chongqing Medical University, Chongqing, China; 2 Department of Laboratory Medicine, the Second Affiliated Hospital of Chongqing Medical University, No.74 Linjiang Road, Chonqing, 400010 Yuzhong District China

**Keywords:** IGF1, Polymorphism, rs2195239, rs2162679, Meta-analysis, Cancer

## Abstract

**Background:**

Many epidemiological studies have suggested that insulin-like growth factor1 (IGF1) gene single-nucleotide polymorphisms (SNPs) may be associated with cancer risk. Among several commonly studied polymorphisms in *IGF1* gene, rs2195239 and rs2162679 attracted many attentions. So we perform a meta-analysis to determine potential associations between *IGF1* rs2195239 and rs2162679 polymorphisms and cancer risk.

**Methods:**

We retrieved relevant articles from the PubMed, Embase, and Web of Science databases up to April 30, 2018. Ultimately, thirteen studies were included in the present meta-analysis, which involved 12,515 cases and 19,651 controls. The odd ratios (ORs) and their 95% confidence intervals (CIs) were pooled to estimate the strength of the associations.

**Results:**

rs2195239 reduces the overall cancer risk in homozygote model, as well as reducing cancer risk in Asian populations in allele, homozygote, and recessive models. No significant relationship was found between rs2195239 and breast or pancreatic cancer risk. rs2162679 reduces the overall cancer risk in allele, homozygote, dominant, and recessive models, as well as reducing cancer risk in Asian populations in allele, homozygote, and recessive models.

**Conclusions:**

*IGF1* rs2195239 and rs2162679 were associated with overall cancer risk based on present studies.

**Electronic supplementary material:**

The online version of this article (10.1186/s12881-019-0749-3) contains supplementary material, which is available to authorized users.

## Background

Insulin-like growth factor1 (IGF1) plays an important role in promoting cell proliferation and inhibiting apoptosis [1]. IGF1 is produced mainly by the liver tissue and is secreted into the circulation [2]. Epidemiological studies have shown that IGF1 is involved in tumor development, high concentrations of serum IGF1 are related to the increased risk of several types of cancer, supporting a potential role on the part of IGF1 in cancer development [3–5].

*IGF1* is located on 12q22–24.1, having no strong linkage disequilibrium with nearby genes [6]. Studies of twins have indicated that 40~60% of the inter-individual variability in IGF1 levels in the circulation depends on genetic factors [7–10]. Single-nucleotide polymorphisms (SNPs) are the important part of genetic variability among individuals. Several *IGF1* SNPs have been reported to be associated with elevated IGF1 levels in the circulation [11–13].

Recently, many studies have described the relationship between the *IGF1* gene rs2195239 and rs2162679 polymorphisms and the risks of various cancers [14–23]. However, the results of the relevant studies are inconsistent. In addition, prior studies regarding the relationship between the rs2195239 and rs2162679 polymorphisms and cancer risk are limited in terms of sample size and thus, statistical power. We performed the present meta-analysis to more precisely describe the relationship between the *IGF1* rs2195239 and rs2162679 polymorphisms and cancer risk.

## Methods

### Publication search

Relevant articles published prior to April 30, 2018 were identified by searching the PubMed, Embase, and Web of Science databases. The key terms used in the search were: “IGF1 or IGF-1 or insulin-like growth factor 1,” “variant or mutation or SNP or polymorphism,” and “cancer or tumor or neoplasm or carcinoma.” Furthermore, we manually checked the reference in the identified articles to identify additional available studies. Our search was restricted to articles written in the English language.

### Inclusion and exclusion criteria

The included articles had to: 1) concern the relationship between the *IGF1* polymorphisms rs2195239 and rs2162679 and cancer risk, 2) be case-control or cohort studies, 3) contain sufficient data on genotype distribution. We excluded comments, editorials, reviews, meta-analyses, and studies lacking sufficient data.

### Data extraction and quality assessment

Two researchers extracted information from all the included studies independently, as well as evaluating the quality of the studies. Controversies were resolved through negotiation. The following data were collected: first author’s name, publication year, type of cancer, ethnicity, method of genotyping, control source, genotype distributions of cases and controls, and the *P*-value for the Hardy-Weinberg equilibrium (HWE) of controls. The quality of the studies was assessed using a quality score form [24] (Additional file 1: Figure S1).

### Statistical analysis

Statistical analyses were applied by using STATA software (Version 12.0, Stata Corporation, College Station, TX, USA). The ORs and 95% CIs were calculated to evaluate the strength of the associations between the *IGF1* rs2195239 and rs2162679 polymorphisms and cancer risk in five genetic models: the allele model (for rs2195239: C vs. G; for 2,162,679: G vs. A), the homozygote model (for rs2195239: CC vs. GG; for 2,162,679: GG vs. AA), the heterozygote model (for rs2195239: GC vs. GG; for 2,162,679: AG vs. AA), the dominant model (for rs2195239: CC + GC vs. GG; for 2,162,679: GG + AG vs. AA), and the recessive model (for rs2195239: CC vs. GC + GG; for 2,162,679: GG vs. AG + AA). Heterogeneity was estimated using a Q test and I^2^ [25]. The fixed-effects model was applied when heterogeneity was absent [26] (*P* > 0.1). Otherwise, the random-effects model were used [27]. The HWE for the controls was calculated using a Chi-squared test. In addition, we carried out stratified analyses according to ethnicity, cancer type, and quality score. Sensitivity analyses were performed to evaluate the stability of the overall analyses excluding a single study at a time. Egger’s tests were applied to assess publication bias [28].

## Results

### Description of search results

**Fig. 1:**
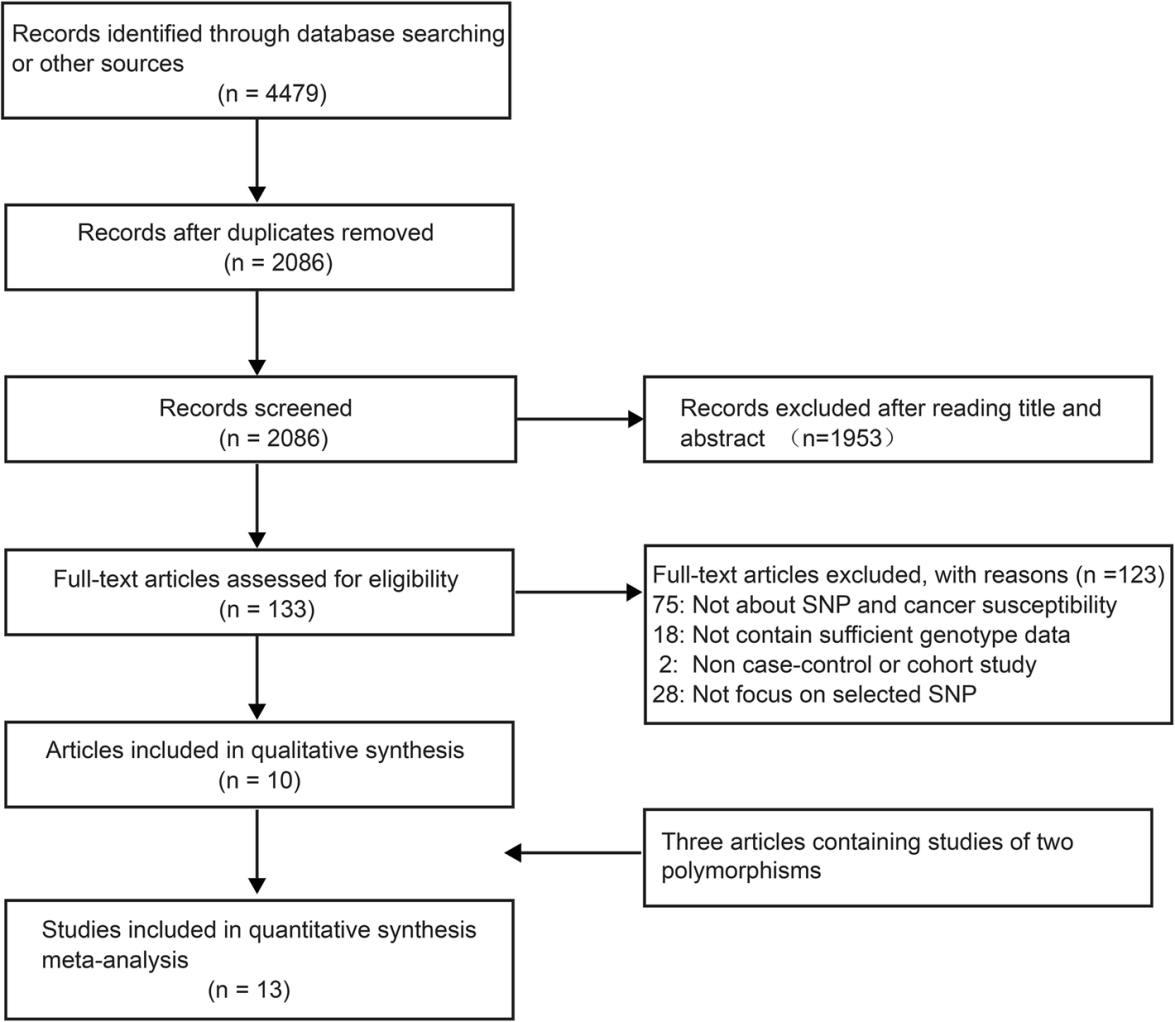
The flow diagram of included/excluded studies

Through searching the databases, a total of 4479 articles were initially obtained. After removing duplicates, 2086 articles were left. After screening the titles and abstracts, 133 articles were retrained for full-text review. Ultimately, ten articles were identified for meta-analysis [14–23] (Fig. 1). Three articles included studies of two *IGF1* polymorphisms [15, 20, 21]. In total, 13 studies from ten articles were included in the current meta-analysis, which involved 12,515 cases and 19,651 controls. The publication year ranged from 2006 to 2016. The characteristics of the studies and the genotype frequencies for cases and controls of rs2195239 and rs2162679 are shown in Tables 1 and 2 respectively.

**Table 1:** Characteristics of the studies included in the meta-analysis for rs2195239

First author	Year	Country/Region	Ethnicity	Cancer type	Genotyping method	Age(y) Case/Control	Control source	Case			Control			HWE
								GG	GC	CC	GG	GC	CC	
Chia [15]	2008	USA	Mix	TGCT	Taqman	≤45/≤45	PB	332	209	32	395	252	50	Y
Patel [17]	2008	USA/Europe	Mix	Breast cancer	Taqman	NR	PB	434	2440	3699	532	3121	4819	Y
Birmann [18]	2009	USA	Caucasian	Multiple myeloma	Taqman	30–75/30–75	PB	9	26	43	11	63	85	Y
Dong [19]	2011	USA	Mix	Pancreatic cancer	MassArray and TaqMan	14–80/14–80	HB	385	270	40	409	260	35	Y
Ennishi [20]	2011	Japan	Asian	Stomach cancer	Taqman	NR	HB	230	346	127	447	703	312	Y
Nakao [21]	2011	Japan	Asian	Pancreatic cancer	Taqman	20–79/20–79	HB	54	95	27	431	673	298	Y
Qian [22]	2011	China	Asian	Breast cancer	Taqman	NR	HB	147	181	75	135	193	75	Y
Shi [23]	2016	Canada	Mix	Breast cancer	Illumina GoldenGate	40–80/40–80	PB	349	267	25	453	301	52	Y

Abbreviations: *TGCT* testicular germ cell tumors, *PB* population-based, *HB* hospital-based, *HWE* Hardy-Weinberg equilibrium, *Y* polymorphisms conformed to HWE in the control group, *N* polymorphisms did not conform to HWE in the control group, *NR* not reported

**Table 2:** Characteristics of the studies included in the meta-analysis for rs2162679

First author	Year	Country/Region	Ethnicity	Cancer type	Genotyping method	Age(y) Case/Control	Control source	Case			Control			HWE
								AA	AG	GG	AA	AG	GG	
Canzian [14]	2006	Europe	Caucasian	Breast cancer	Taqman	35–69/35–69	PB	570	212	19	1060	446	61	Y
Chia [15]	2008	USA	Mix	TGCT	Taqman	≤45/≤45	PB	408	149	16	492	188	23	Y
Lonn [16]	2008	USA	Mix	Brain tumor	Taqman	≥18/≥18	HB	313	97	10	300	103	9	Y
Ennishi [20]	2011	Japan	Asian	Stomach cancer	Taqman	NR	HB	330	293	80	608	637	217	N
Nakao [21]	2011	Japan	Asian	Pancreatic cancer	Taqman	20–79/20–79	HB	70	87	19	580	613	209	N

Abbreviations: *TGCT* testicular germ cell tumors, *PB* population-based, *HB* hospital-based, *HWE* Hardy-Weinberg equilibrium, *Y* polymorphisms conformed to HWE in the control group, *N* polymorphisms did not conform to HWE in the control group

### Meta-analysis

The relationship between the IGF1 *gene* rs2195239 and rs2162679 polymorphisms and cancer risk were evaluated using ORs and 95% CI in the allele, homozygote, heterozygote, dominant, and recessive models. We also conducted stratified analyses according to ethnicity, cancer type, and score. Only results synthesized from no fewer than two studies are shown.

**Fig. 2:**
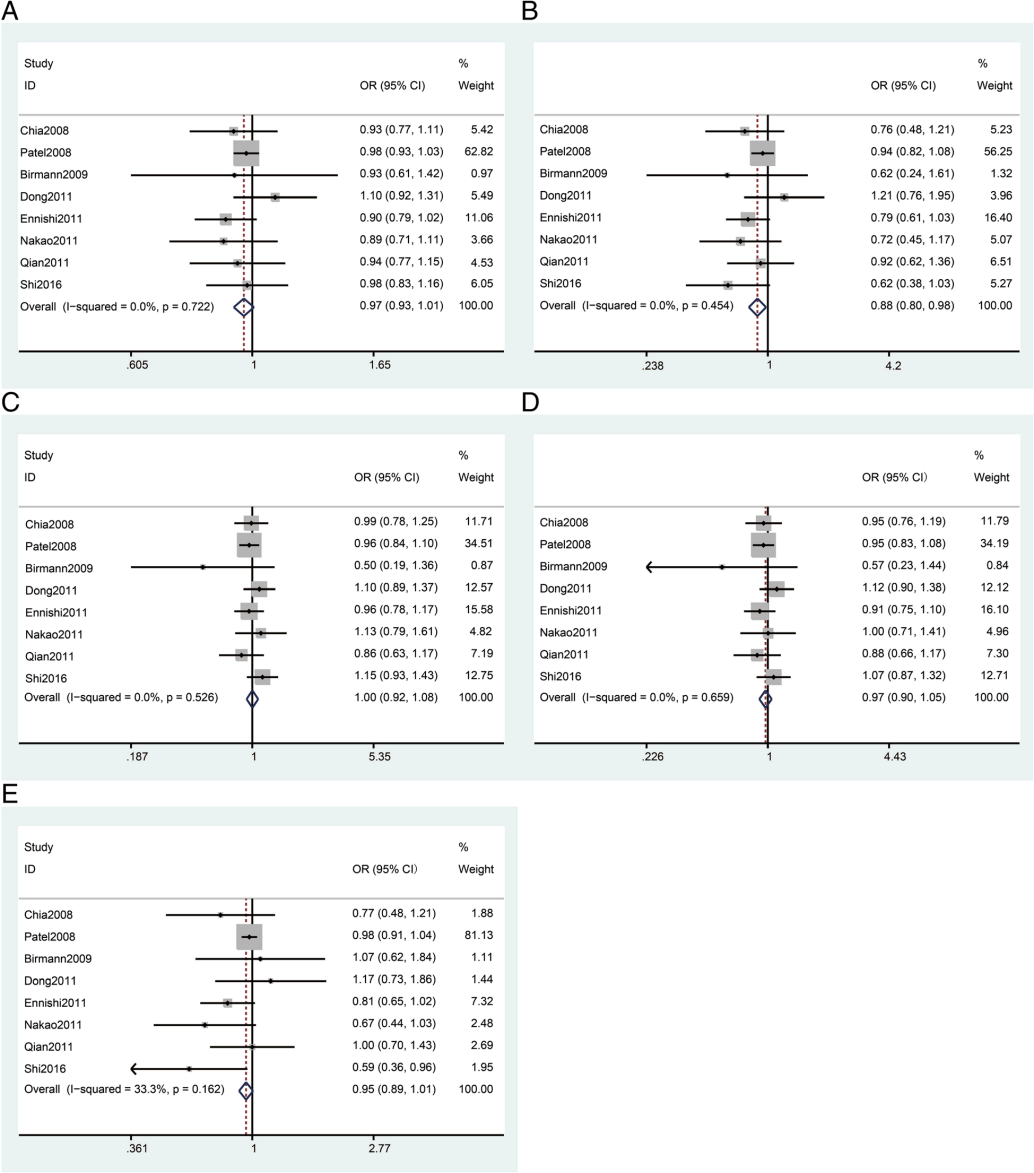
Meta-analysis of rs2195239 polymorphism and cancer risk. **a**: allele model; **b**: homozygous model; **c**: heterozygous model; **d**: dominant model; **e**: recessive model. The squares and horizontal lines correspond to the study specific OR and 95% CI. The area of the squares reflects the weight. The diamond represents the summary OR and 95% CI. The fixed-effects model was used

There were a total of 9842 cases and 14,105 controls included from eight studies regarding the rs2195239 polymorphism. In overall analysis, rs2195239 was shown to be significantly associated with reduced cancer risk (*n* = 8, Table 3 and Fig. 2, CC vs. GG: OR = 0.88, 95% Cl = 0.80–0.98, *P* = 0.018). In the analyses stratified by ethnicity, rs2195239 was shown to significantly reduce cancer risk in Asian populations (*n* = 3, Table 3, C vs. G: OR = 0.91, 95% Cl = 0.82–1.00, *P* = 0.044; CC vs. GG: OR = 0.81, 95% Cl = 0.66–0.99, *P* = 0.035; CC vs. GC + GG: OR = 0.83, 95%Cl = 0.69–0.98, *P* = 0.031). In the analyses stratified by cancer type, no significant relationship between rs2195239 and breast (n = 3, Table 3) or pancreatic cancer (*n* = 2, Table 3) risk was found. The scores for all of the studies regarding rs2195239 are no less than twelve.

**Table 3:** Meta-analysis of the association between rs2195239 and rs2162679 polymorphisms and cancer risk

Subgroup	*N*o.	Allele model			Homozygote model			Heterozygote model			Dominant model			Recessive model		
		OR(95% Cl)	*P*_*OR*_	*P*_*h*_	OR(95% Cl)	*P*_*OR*_	*P*_*h*_	OR(95% Cl)	*P*_*OR*_	*P*_*h*_	OR(95% Cl)	*P*_*OR*_	*P*_*h*_	OR(95% Cl)	*P*_*OR*_	*P*_*h*_
rs2195239		C vs. G			CC vs. GG			GC vs. GG			CC + GC vs. GG			CC vs. GC + GG		
Overall	8	0.97(0.93–1.01)	0.103	0.722	**0.88(0.80–0.98)**	**0.018**	0.454	1.00 (0.93–1.10)	0.972	0.526	0.97(0.90–1.05)	0.466	0.659	0.95 (0.90–1.01)	0.083	0.162
Asian	3	**0.91 (0.82–1.00)**	**0.044**	0.906	**0.81 (0.66–0.99)**	**0.035**	0.731	0.96 (0.83–1.12)	0.616	0.534	0.92 (0.79–1.06)	0.232	0.831	**0.83 (0.69–0.98)**	**0.031**	0.362
Breast cancer	3	0.97(0.93–1.02)	0.282	0.939	0.91 (0.81–1.03)	0.152	0.294	0.99 (0.89–1.10)	0.866	0.238	0.97(0.87–1.07)	0.536	0.474	0.97(0.91–1.03)	0.308	0.131
Pancreatic cancer	2	1.02 (0.89–1.16)	0.833	0.139	0.94 (0.67–1.31)	0.710	0.134	1.11 (0.92–1.34)	0.275	0.921	1.08 (0.91–1.30)	0.466	0.600	0.88(0.51–1.51)^*^	0.640	0.087
rs2162679		G vs. A			GG vs. AA			AG vs. AA			GG + AG vs. AA			GG vs. AG + AA		
Overall	5	**0.87 (0.80–0.94)**	**0.001**	0.693	**0.70 (0.57–0.87)**	**0.001**	0.787	0.91 (0.82–1.02)	0.209	0.556	**0.88(0.79–0.97)**	**0.011**	0.578	**0.73 (0.60–0.89)**	**0.002**	0.817
Asian	2	**0.85 (0.76–0.96)**	**0.007**	0.302	**0.70 (0.54–0.90)**	**0.005**	0.737	0.97(0.71–1.33)^*^	0.845	0.096	0.86 (0.74–1.01)	0.065	0.131	**0.73 (0.57–0.92)**	**0.009**	0.824
Quality score ≥ 12	2	**0.87 (0.77–0.99)**	**0.037**	0.389	**0.67 (0.44–1.00)**	**0.049**	0.385	0.91 (0.78–1.06)	0.225	0.629	0.88(0.76–1.02)	0.093	0.492	0.68 (0.46–1.02)	0.064	0.412

Abbreviations: *OR* odds ratio, 95% CI 95% confidence interval, *P*_*OR*_ pool *P* value, *P*_*h*_ *P* value of heterogeneity test, *indicates that the OR, 95% Cl, and corresponding *P*_*OR*_ were calculated based on the random-effects model; otherwise, the fixed-effects model was used. Bold values are statistically significant (*P*_*OR*_ < 0.05)

**Fig. 3:**
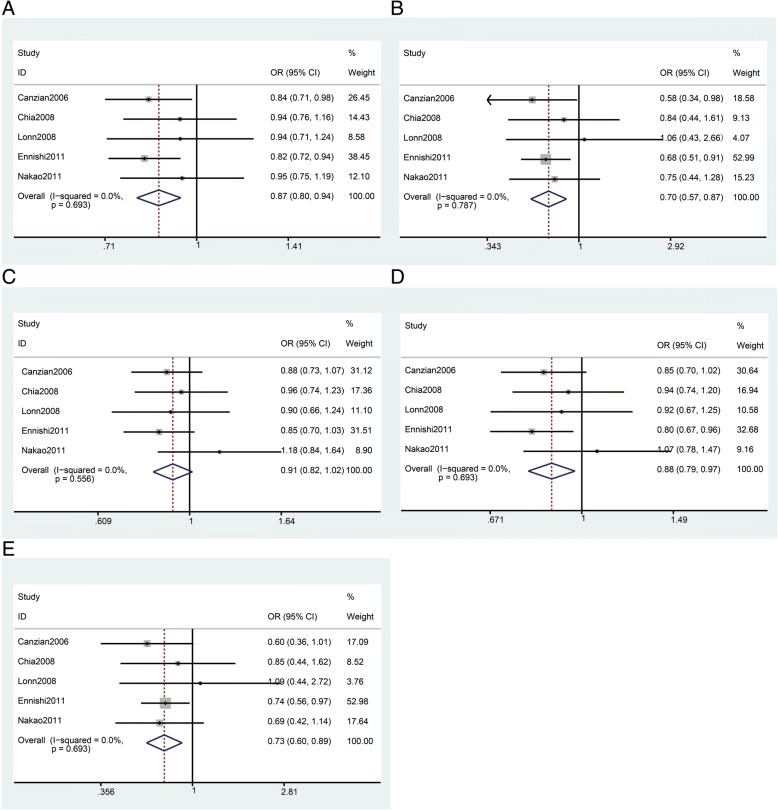
Meta-analysis of rs2162679 polymorphism and cancer risk. **a**: allele model; **b**: homozygous model; **c**: heterozygous model; **d**: dominant model; **e**: recessive model. The squares and horizontal lines correspond to the study specific OR and 95% CI. The area of the squares reflects the weight. The diamond represents the summary OR and 95% CI. The fixed-effects model was used

There were a total of 2673 cases and 5546 controls from five studies regarding the rs2162679 polymorphism. In overall analysis, rs2162679 was shown to be significantly associated with reduced cancer risk (*n* = 5, Table 3 and Fig. 3, G vs. A: OR = 0.87, 95% Cl = 0.80–0.94, *P* = 0.001; GG vs. AA: OR = 0.70, 95% Cl, =0.57–0.87, P = 0.001; GG + AG vs. AA: OR = 0.88, 95%Cl = 0.79–0.97, *P* = 0.011; GG vs. AG + AA: OR = 0.73, 95% Cl = 0.60–0.89, *P* = 0.002). In the analyses stratified by ethnicity, we found that rs2162679 was shown to significantly reduce cancer risk in Asian populations (*n* = 2, Table 3 and Fig. 2, G vs. A: OR = 0.85, 95% Cl = 0.76–0.96, *P* = 0.007; GG vs. AA: OR = 0.70, 95% Cl = 0.54–0.90, *P* = 0.005; GG vs. AG + AA: OR = 0.73, 95% Cl = 0.57–0.92, *P* = 0.009). The results synthesized from these studies, which score no less than twelve, showed that rs2162679 reduces cancer risk in the allele and homozygote models (*n* = 2,Table 3), indicating that the results for rs2162679 are relatively stable in these models.

### Sensitivity analysis

**Fig. 4:**
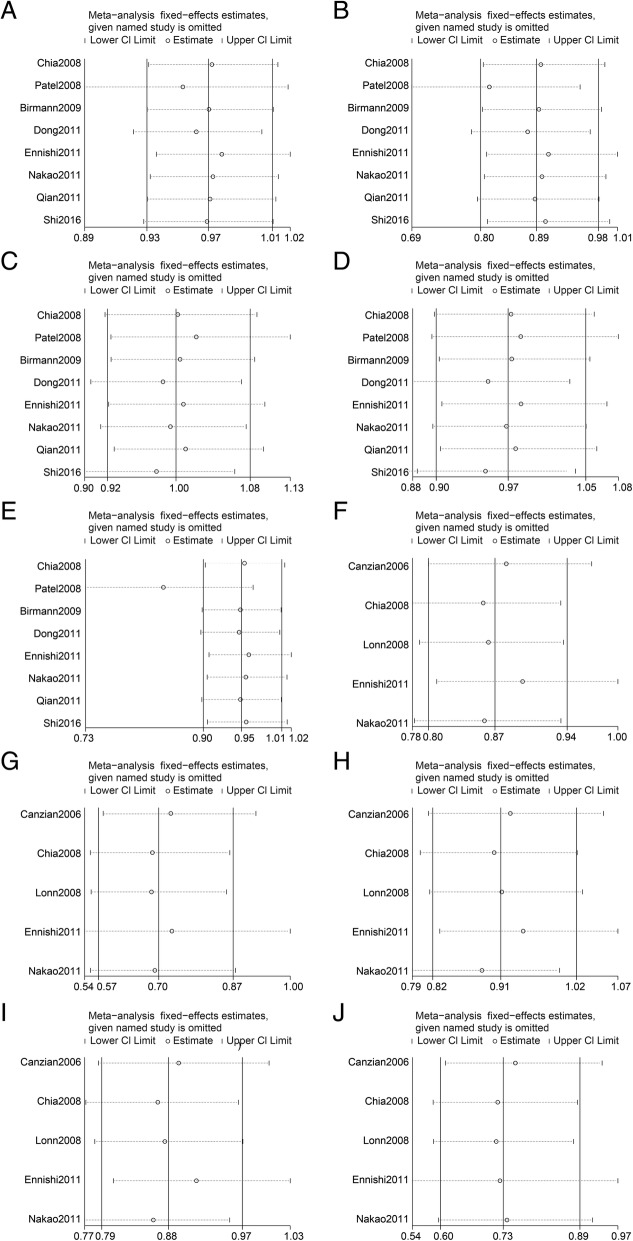
Sensitivity analyses between rs2195239 and rs2162679 polymorphisms and cancer risk. A-E Sensitivity analyses for rs2195239, **a**: allele model; **b**: homozygous model; **c**: heterozygous model; **d**: dominant model; **e**: recessive model**. f**-**j** Sensitivity analyses for rs2162679, **f**: allele model; **g**: homozygous model; **h**: heterozygous model; **i**: dominant model; **j**: recessive model. The fixed-effects model was used

A sensitivity analysis was conducted by excluding a single study at a time. The sensitivity analysis for rs2195239 suggests that excluding the study by Ennishi et al. would have led to a different result in the homozygote model as compared with the results of the overall analysis (Fig. 4 and Additional file 1: Table S2), and excluding the study by Patel et al. would have led to a different result in the recessive model as compared with the results of the overall analysis (Fig. 4 and Additional file 1: Table S2). The sensitivity analysis regarding rs2162679 suggests that excluding the study by Nakao et al. would have led to a different result in the heterozygote model, and excluding the study by Canzian et al., or Ennishi et al. would have led to a different result in the dominant model as compared with the results in the overall analysis (Fig. 4 and Additional file 1: Table S2).

The instability of the sensitivity analyses indicated that the number of studies included in our meta-analysis was not sufficient, and the conclusions drawn from the present meta-analysis should be verified in the future.

### Publication bias

Egger’s tests were applied to detect publication bias. We did not detect publication bias for rs2195239 (Table 4). However, for rs2162679, there was publication bias in the allele (Egger’s test *P* = 0.020) and dominant models (Egger’s test *P* = 0.046, Table 4).

**Table 4:** Publication bias analysis

Polymorphism	Genetic model	Egger’s test		
		t	95% Cl	*P*
rs2195239	C vs. G	−0.58	−1.599-0.982	0.580
	CC vs. GG	−1.46	−2.322~0.586	0.194
	GC vs. GG	−0.66	−2.785~1.596	0.531
	CC + GC vs. GG	−0.76	−2.581~1.364	0.479
	CC vs. GC + GG	−1.48	− 2.177~0.534	0.189
rs2162679	G vs. A	4.55	0.750–4.235	0.020
	GG vs. AA	1.38	−1.276~3.225	0.262
	AG vs. AA	2.14	−1.298~6.601	0.122
	GG + AG vs. AA	3.28	0.088~5.706	0.046
	GG vs. AG + AA	0.71	−1.883~2.973	0.527

## Discussion

IGF1 stimulates cell proliferation, decreases apoptosis, and is thus involved in cancer development [4]. There have been many well-designed cohort studies, such as the BPC3 cohort, and case-control studies regarding *IGF1* polymorphisms and cancer risk in the past few years [29–31]. We conducted this meta-analysis to summarize the results of these studies regarding the *IGF1* gene rs2195239 and rs2162679 polymorphisms and cancer risk.

Several polymorphisms, including rs6214, rs6220, rs5742714, rs1549593, 2,373,722, 10,735,380, 12,821,878, rs2195239, rs2162679, rs35767, rs5742612, and rs7965399, have been reported to be related to disease occurrence [15, 18, 20–23, 32–34]. Some of these important polymorphisms, such as rs6214, rs6220, and rs5742714,are located in the 3’UTR region of *IGF1*, while others, such as rs1549593, 2,373,722, 10,735,380, 12,821,878, rs2195239, and rs2162679, are located in the intron of *IGF1*. And other polymorphisms, such as rs35767, rs7965399, and rs5742612, are located in other regions of *IGF1.* Among the polymorphisms located in the intron of *IGF1*, we chose rs2195239 and 2,162,679 because they have been reported to be related to cancer risk in many studies, and in the 1000 Genomes Project Phase 3, the minor allele frequencies (MAFs) of the SNPs were shown to be higher than 20% among most of the populations (Additional file 1: Table S3). There was no close linkage disequilibrium (LD) between rs2162679 and other *IGF1* polymorphisms in several populations (data not shown), and there was no close LD between rs2162679 and rs2195239 (Additional file 1: Figure S1). Life is a piece of melodious music composed of A/T/C/G notes, and we always want to explore which notes will affect the tone of the entire musical piece, for example, by causing cancer.

It has been reported that rs2195239 reduces relapse risk in stomach cancer patients after curative gastrectomy [35]. Also, rs2195239 has been shown to have a significant association with the pathological progression of childhood IgA nephropathy [36]. In our meta-analysis, we found that rs2195239 reduced cancer risk in overall analysis, as well as reducing the risk of cancer in Asian populations.

The rs2162679 GG genotype has been reported to be associated with a reduced risk of breast cancer, and this effect is more significant in the patients who were diagnosed before turning 55 years old [14]. In our meta-analysis, we found that rs2162679 reduced cancer risk in overall analysis and also reduced cancer risk in Asian populations.

The *IGF1* SNPs affect cancer susceptibility mainly by influencing the serum levels of IGF1. The rs2195239 polymorphism has a reported association with significantly decreased IGF1 levels in the circulation [17]. The effect of rs2162679 on serum IGF1 levels has not been reported previously, and relevant studies of the biological functions of these two polymorphisms are relatively limited. Some studies have been conducted on the biological functions of other *IGF1* SNPs that appear to affect cancer susceptibility. For example, rs1520220 may influence the expression of circulating IGF1 by altering the secondary structure of the RNA or DNA [37, 38]. Previously, rs5742714 was observed to create a microRNA binding site for hsa-mir-580 [33]. The possibility of linkages between some SNPs and functional alleles at exons had also been suggested, and this also could influence the serum levels of IGF1 [39]. The biological functions of rs2195239 and rs2162679 and the mechanisms by which they affect cancer susceptibility should be explored further in future studies.

Many researchers now hold that the studies having less than 100 patients do not have sufficient power to reveal genetic associations. We attempted to exclude the studies less than 100 subjects (Birmann et al., 2009) and found that the conclusions remained the same (Additional file 1: Table S4). We noted that the genotyping frequency reported by Patel et al. 2008 is very different from that reported in the other studies. Specifically, the CC genotype seems to have a much higher frequency in Patel et al. as compared to the other studies. We believe that there are two potential reasons for this. One possibility is that this difference in genotype frequency was caused by ethnic differences. Another possibility is that a genotyping error occurred. We removed Patel et al.’s study and conducted a meta-analysis of the other studies (Additional file 1: Table S4). We found that the conclusions remained fundamentally the same. Because Patel’s study was drawn from a huge cohort study, BP3, we decided to retain these data in the meta-analysis, but this difference in genotyping frequency does warrant caution.

The present meta-analysis has several limitations. First, the number of articles included in this study was limited. In the stratified analysis, pooled studies were not performed for a specific ethnic group containing only one single study, such as Caucasians and rs2162679. Secondly, the cancer types included in the study were limited, and this may have introduced bias into the results. For each SNP, the study only included five types of cancers; therefore, confirmation of whether the conclusions drawn from these types of cancer reflect the true relationship between this SNP and the overall cancer risk will require further investigation. In the future, we should verify these conclusions by examining additional types of cancer. Thirdly, the meta-analysis did not consider the potential determinants factors such as gender, age, and alcohol and tobacco intake. Finally, the sample size for the publications included in this study varied substantially. In several studies, the genotype distribution in control groups did not conform to HWE. Moreover, for rs2162679, publication bias was detected, and for both rs2195239 and rs2162679, the results of the sensitivity analyses were unstable. For these reasons, the findings should be interpreted with caution.

## Conclusion

In conclusion, meta-analysis suggests that rs2195239 reduces the overall cancer risk in homozygote model, as well as reducing cancer risk in Asian populations in allele, homozygote, and recessive models. No significant relationship was found between rs2195239 and breast or pancreatic cancer risk. rs2162679 reduces the overall cancer risk in allele, homozygote, dominant, and recessive models, as well as reducing cancer risk in Asian populations in allele, homozygote, and recessive models. However, considering the limitations of our meta-analysis and the publication bias between studies, the associations based on present studies should be verified with more studies in the future.

## Additional file

Additional file 1: **Table S1.** Quality score assessment. **Table S2.** Sensitivity analyses for rs2195239 and rs2162679 polymorphisms and cancer risk. **Table S3.** MAFs of rs2195239 (genomic position: chromosome12: 102462924) and rs2162679 (genomic position: Chromosome12: 102477481) and polymorphisms in the populations from the 1000 Genomes Project Phase 3. **Table S4.** Meta-analysis of the association between rs2195239 polymorphism and cancer risk, omitting the study of Patel or Birmann. **Table S5.** The OMIM numbers for important genes and pathogenic conditions in this study. **Figure S1.** Linkage disequilibrium analyses for IGF1 rs2195239 and rs2162679 polymorphisms in populations from the 1000 Genomes Project Phase 3. (ZIP 1155 kb)

## Abbreviations

3’-UTR: three prime untranslated region
CI: Confidence interval
HB: Hospital-based
HWE: Hardy-Weinberg equilibrium
IGF1: Insulin-like growth factor 1
LD: Linkage disequilibrium
MAF: Minor allele frequency
OMIM: Online Mendelian Inheritance in Man
OR: Odds ratio
PB: Population-based
SNP: Single nucleotide polymorphism
TGCT: Testicular germ cell tumors
