# Supplementary material for: Association between the insulin-like growth factor 1 gene rs2195239 and rs2162679 polymorphisms and cancer risk: a meta-analysis
Source: BMC Med Genet. 2019 Jan 17;20:17. doi: 10.1186/s12881-019-0749-3 (PMC6337782; doi:10.1186/s12881-019-0749-3)
Supplement: Supplementary file 1 — Table S1. Quality score assessment. Table S2. Sensitivity analyses for rs2195239 and rs2162679 polymorphisms and cancer risk. Table S3. MAFs of rs2195239 (genomic position: chromosome12: 102462924) and rs2162679 (genomic position: Chromosome12: 102477481) and polymorphisms in the populations from the 1000 Genomes Project Phase 3. Table S4. Meta-analysis of the association between rs2195239 polymorphism and cancer risk, omitting the study of Patel or Birmann. Table S5. The OMIM numbers for important genes and pathogenic conditions in this study. Figure S1. Linkage disequilibrium analyses for IGF1 rs2195239 and rs2162679 polymorphisms in populations from the 1000 Genomes Project Phase 3. (ZIP 1155 kb) [file 12881_2019_749_MOESM1_ESM.zip › Supplementary Tables and Supplementary Figure legend.docx]

**Scale for quality assessment criterion [24]**

|  | Criterion | Score |
| --- | --- | --- |
| **A** | **Source of cases** |  |
|  | Selected from population or cancer registry | 3 |
|  | Selected from hospital | 2 |
|  | Selected from pathology archives, but without description | 1 |
|  | Not described | 0 |
| **B** | **Source of controls** |  |
|  | Population-based | 3 |
|  | Blood donors or volunteers | 2 |
|  | Hospital-based (cancer-free patients) | 1 |
|  | Not described | 0 |
| **C** | **Specimens used for determining genotypes** |  |
|  | White blood cells or normal tissues | 3 |
|  | Not mentioned | 2 |
|  | Tumor tissues or exfoliated cells of tissue | 0 |
| **D** | **Hardy–Weinberg equilibrium in controls** |  |
|  | Hardy–Weinberg equilibrium | 3 |
|  | Hardy–Weinberg disequilibrium | 0 |
| **E** | **Total sample size** |  |
|  | ≥1,000 | 3 |
|  | ≥500 and <1,000 | 2 |
|  | ≥200 and <500 | 1 |
|  | <200 | 0 |

**Table S1: Quality score assessment**

| **rs2195239** | **A** | **B** | **C** | **D** | **E** | **Score** |
| --- | --- | --- | --- | --- | --- | --- |
| Chia2008 | 3 | 3 | 3 | 3 | 3 | 15 |
| Patel2008 | 3 | 3 | 3 | 3 | 3 | 15 |
| Birmann2009 | 3 | 3 | 3 | 3 | 1 | 13 |
| Dong2011 | 2 | 1 | 3 | 3 | 3 | 12 |
| Ennishi2011 | 2 | 1 | 3 | 3 | 3 | 12 |
| Nakao2011 | 2 | 1 | 3 | 3 | 3 | 12 |
| Qian2011 | 2 | 1 | 3 | 3 | 3 | 12 |
| Shi2016 | 3 | 3 | 3 | 3 | 3 | 15 |
| **rs2162679** |  |  |  |  |  |  |
| Canzian2006 | 3 | 3 | 3 | 3 | 3 | 15 |
| Chia2008 | 3 | 3 | 3 | 3 | 3 | 15 |
| Lonn2008 | 2 | 1 | 3 | 3 | 2 | 11 |
| Ennishi2011 | 2 | 1 | 3 | 0 | 3 | 9 |
| Nakao2011 | 2 | 1 | 3 | 0 | 3 | 9 |

A-E represents the corresponding criterion in the table of scale for quality assessment criterion.

**Table S2: Sensitivity analyses for rs2195239 and rs2162679 polymorphisms and cancer risk**

| **SNP** | **Comparison** | **Study omitted** | **Estimate** | **[95% Confident Interval]** | **Effect model** |
| --- | --- | --- | --- | --- | --- |
| **rs2195239** | **C vs. G** | Chia2008 | 0.968 | 0.927-1.011 | Fixed |
|  |  | Patel2008 | 0.949 | 0.886-1.017 |  |
|  |  | Birmann2009 | 0.966 | 0.926-1.008 |  |
|  |  | Dong2011 | 0.958 | 0.918-1.000 |  |
|  |  | Ennishi2011 | 0.974 | 0.932-1.019 |  |
|  |  | Nakao2011 | 0.969 | 0.928-1.011 |  |
|  |  | Qian2011 | 0.967 | 0.926-1.009 |  |
|  |  | Shi2016 | 0.965 | 0.924-1.008 |  |
|  |  | Combined | 0.966 | 0.926-1.007 |  |
|  | **CC vs. GG** | Chia2008 | 0.892 | 0.803-0.991 | Fixed |
|  |  | Patel2008 | 0.813 | 0.693-0.953 |  |
|  |  | Birmann2009 | 0.889 | 0.802-0.986 |  |
|  |  | Dong2011 | 0.872 | 0.785-0.968 |  |
|  |  | Ennishi2011 | **0.904** | **0.809-1.010** |  |
|  |  | Nakao2011 | 0.894 | 0.805-0.993 |  |
|  |  | Qian2011 | 0.883 | 0.794-0.982 |  |
|  |  | Shi2016 | 0.899 | 0.810-0.999 |  |
|  |  | Combined | 0.885 | 0.799-0.981 |  |
|  | **GC vs. GG** | Chia2008 | 1.003 | 0.921-1.092 | Fixed |
|  |  | Patel2008 | 1.024 | 0.928-1.130 |  |
|  |  | Birmann2009 | 1.006 | 0.928-1.090 |  |
|  |  | Dong2011 | 0.987 | 0.905-1.075 |  |
|  |  | Ennishi2011 | 1.009 | 0.925-1.101 |  |
|  |  | Nakao2011 | 0.995 | 0.917-1.080 |  |
|  |  | Qian2011 | 1.012 | 0.932-1.099 |  |
|  |  | Shi2016 | 0.979 | 0.898-1.067 |  |
|  |  | Combined | 1.001 | 0.924-1.084 |  |
|  | **CC+GC vs. GG** | Chia2008 | 0.975 | 0.899-1.057 | Fixed |
|  |  | Patel2008 | 0.984 | 0.896-1.081 |  |
|  |  | Birmann2009 | 0.975 | 0.904-1.053 |  |
|  |  | Dong2011 | 0.952 | 0.877-1.033 |  |
|  |  | Ennishi2011 | 0.985 | 0.906-1.070 |  |
|  |  | Nakao2011 | 0.970 | 0.897-1.049 |  |
|  |  | Qian2011 | 0.979 | 0.905-1.060 |  |
|  |  | Shi2016 | 0.957 | 0.882-1.039 |  |
|  |  | Combined | 0.972 | 0.900-1.049 |  |
|  | **CC vs. GC+GG** | Chia2008 | 0.954 | 0.899-1.013 | Fixed |
|  |  | Patel2008 | **0.839** | **0.728-0.967** |  |
|  |  | Birmann2009 | 0.949 | 0.894-1.007 |  |
|  |  | Dong2011 | 0.947 | 0.893-1.006 |  |
|  |  | Ennishi2011 | 0.961 | 0.904-1.022 |  |
|  |  | Nakao2011 | 0.957 | 0.902-1.016 |  |
|  |  | Qian2011 | 0.949 | 0.894-1.008 |  |
|  |  | Shi2016 | 0.957 | 0.902-1.016 |  |
|  |  | Combined | 0.951 | 0.896-1.008 |  |
| **rs2162679** | **G vs. A** | Canzian2006 | 0.880 | 0.800-0.968 | Fixed |
|  |  | Chia2008 | 0.856 | 0.783-0.936 |  |
|  |  | Lonn2008 | 0.861 | 0.790-0.939 |  |
|  |  | Ennishi2011 | 0.897 | 0.808-0.995 |  |
|  |  | Nakao2011 | 0.857 | 0.785-0.936 |  |
|  |  | Combined | 0.868 | 0.799-0.943 |  |
|  | **GG vs. AA** | Canzian2006 | 0.731 | 0.581-0.919 | Fixed |
|  |  | Chia2008 | 0.690 | 0.553-0.862 |  |
|  |  | Lonn2008 | 0.688 | 0.555-0.854 |  |
|  |  | Ennishi2011 | 0.733 | 0.540-0.995 |  |
|  |  | Nakao2011 | 0.696 | 0.553-0.874 |  |
|  |  | Combined | 0.704 | 0.571-0.869 |  |
|  | **AG vs. AA** | Canzian2006 | 0.926 | 0.815-1.052 | Fixed |
|  |  | Chia2008 | 0.904 | 0.804-1.016 |  |
|  |  | Lonn2008 | 0.914 | 0.817-1.023 |  |
|  |  | Ennishi2011 | 0.943 | 0.830-1.072 |  |
|  |  | Nakao2011 | **0.887** | **0.793-0.993** |  |
|  |  | Combined | 0.913 | 0.821-1. 015 |  |
|  | **GG+AG vs. AA** | Canzian2006 | **0.890** | **0.788-1.005** | Fixed |
|  |  | Chia2008 | 0.863 | 0.772-0.966 |  |
|  |  | Lonn2008 | 0.872 | 0.783-0.972 |  |
|  |  | Ennishi2011 | **0.912** | **0.807-1.032** |  |
|  |  | Nakao2011 | 0.858 | 0.771-0.955 |  |
|  |  | Combined | 0.877 | 0.792-0.971 |  |
|  | **GG vs. AG+AA** | Canzian2006 | 0.756 | 0.608-0.941 | Fixed |
|  |  | Chia2008 | 0.719 | 0.582-0.889 |  |
|  |  | Lonn2008 | 0.716 | 0.582-0.880 |  |
|  |  | Ennishi2011 | 0.724 | 0.537-0.975 |  |
|  |  | Nakao2011 | 0.739 | 0.593-0.921 |  |
|  |  | Combined | 0.731 | 0.597-0.894 |  |

**Table S3: MAFs of rs2195239 (genomic position: chromosome12: 102462924) and rs2162679 (genomic position: Chromosome12: 102477481) and polymorphisms in the populations from the 1000 Genomes Project Phase 3**

| Populations | rs2195239 | rs2162679 |
| --- | --- | --- |
| ACB | 0.448 | 0.458 |
| ASW | 0.459 | 0.352 |
| BEB | 0.32 | 0.314 |
| CDX | 0.43 | 0.43 |
| CEU | 0.242 | 0.116 |
| CHB | 0.422 | 0.35 |
| CHS | 0.443 | 0.343 |
| CLM | 0.181 | 0.207 |
| ESN | 0.369 | 0.384 |
| FIN | 0.333 | 0.197 |
| GBR | 0.209 | 0.203 |
| GIH | 0.243 | 0.325 |
| GWD | 0.31 | 0.496 |
| IBS | 0.206 | 0.159 |
| ITU | 0.275 | 0.255 |
| JPT | 0.404 | 0.322 |
| KHV | 0.434 | 0.379 |
| LWK | 0.5 | 0.439 |
| MSL | 0.382 | 0.441 |
| MXL | 0.227 | 0.258 |
| PEL | 0.188 | 0.259 |
| PJL | 0.312 | 0.234 |
| PUR | 0.231 | 0.231 |
| STU | 0.294 | 0.245 |
| TSI | 0.159 | 0.126 |
| YRI | 0.37 | 0.472 |

MAFs: minor allele frequencies; ACB: African Carribbeans in Barbados; ASW: Americans of African Ancestry in SW USA; BEB: Bengali from Bangladesh; CDX: Chinese Dai in Xi -shuangbanna, China; CEU: Utah Residents with Northern and Western European Ancestry; CHB: Han Chinese in Beijing, China; CHS: Southern Han Chinese; CLM: Colombians from Medellin, Colombia; ESN: Esan in Nigeria; FIN: Finnish in Finland; GBR: British in England and Scotland; GIH: Gujarati Indian from Houston, Texas; GWD: Gambian in Western Division in the Gambia; IBS: Iberian Population in Spain; ITU: Indian Telugu from the UK; JPT: Japanese in Tokyo, Japan; KHV: Kinh in Ho Chi Minh City, Vietnam; LWK: Luhya in Webuye, Kenya; MSL: Mende in Sierra Leone; MXL: Mexican Ancestry in Los Angeles USA; PEL: Peruvians from Lima, Peru; PJL:  Punjabi from Lahore, Pakistan; PUR: Puerto Rican in Puerto Rico; STU: Sri Lankan Tamil from the UK; TSI: Toscani in Italia; YRI: Yoruba in Ibadan, Nigeria; N/A: MAF not available in these populations in 1000 Genomes.

**Table S4: Meta-analysis of the association between rs2195239 polymorphism and cancer risk, omitting the study of Patel or Birmann**

| **Group** | **No.** | **Allele model** | | | **Homozygote model** | | | **Heterozygote model** | | | **Dominant model** | | | **Recessive model** | | |
| --- | --- | --- | --- | --- | --- | --- | --- | --- | --- | --- | --- | --- | --- | --- | --- | --- |
|  |  | **OR(95%Cl)** | ***P_OR_*** | ***P_h_*** | **OR(95%Cl)** | ***P_OR_*** | ***P_h_*** | **OR(95%Cl)** | ***P_OR_*** | ***P_h_*** | **OR(95%Cl)** | ***P_OR_*** | ***P_h_*** | **OR(95%Cl)** | ***P_OR_*** | ***P_h_*** |
| **rs2195239** |  | **C vs. G** | | | **CC vs. GG** | | | **GC vs. GG** | | | **CC+GC vs. GG** | | | **CC vs. GC+GG** | | |
| **Overall** | 8 | 0.97(0.93-1.01) | 0.103 | 0.722 | **0.88(0.80-0.98)** | **0.018** | 0.454 | 1.00(0.93-1.10) | 0.972 | 0.526 | 0.97(0.90-1.05) | 0.466 | 0.659 | 0.95(0.90-1.01) | 0.083 | 0.162 |
| **Delete Patel** | 7 | 0.95(0.89-1.02) | 0.139 | 0.661 | **0.81(0.69-0.95)** | **0.010** | 0.563 | 1.02(0.93-1.13) | 0.634 | 0.478 | 0.99(0.90-1.08) | 0.746 | 0.570 | **0.84(0.73-0.96)** | **0.012** | 0.328 |
| **Delete Birmann** | 7 | 0.97(0.93-1.01) | 0.108 | 0.615 | **0.89(0.80-0.98)** | **0.023** | 0.400 | 1.01(0.93-1.09) | 0.888 | 0.640 | 0.98(0.90-1.05) | 0.524 | 0.714 | 0.95(0.89-1.01) | 0.076 | 0.112 |

**Table S5: The OMIM numbers for important genes and pathogenic conditions in this study**

| Gene or pathogenic conditions | OMIM number |
| --- | --- |
| IGF1 | 147440 |
| TGCT | 273300 |
| Breast cancer | 114480 |
| Multiple myeloma | 254500 |
| Pancreatic cancer | 260350 |
| Stomach cancer | 613659 |

Abbreviations: OMIM, Online Mendelian Inheritance in Man; TGCT, testicular germ cell tumors.

**Supplementary figure legend**

**Figure S1: Linkage disequilibrium analyses for IGF1 rs2195239 and rs2162679 polymorphisms in populations from the 1000 Genomes Project Phase 3.**

A. ASW; B. CEU; C. CHB D.CLM; E.GBR; F.GIH; G.GWD; H.MXL; I. PJL; J. STU.

ASW: Americans of African Ancestry in SW USA; CEU: Utah Residents with Northern and Western European Ancestry; CHB: Han Chinese in Beijing, China; CLM: Colombians from Medellin, Colombia; GBR: British in England and Scotland; GIH: Gujarati Indian from Houston, Texas; GWD: Gambian in Western Division in the Gambia; MXL: Mexican Ancestry in Los Angeles USA; PJL: Punjabi from Lahore, Pakistan.
