# Supplementary figures and images for: Association between the insulin-like growth factor 1 gene rs2195239 and rs2162679 polymorphisms and cancer risk: a meta-analysis
Source: BMC Med Genet. 2019 Jan 17;20:17. doi: 10.1186/s12881-019-0749-3 (PMC6337782; doi:10.1186/s12881-019-0749-3)

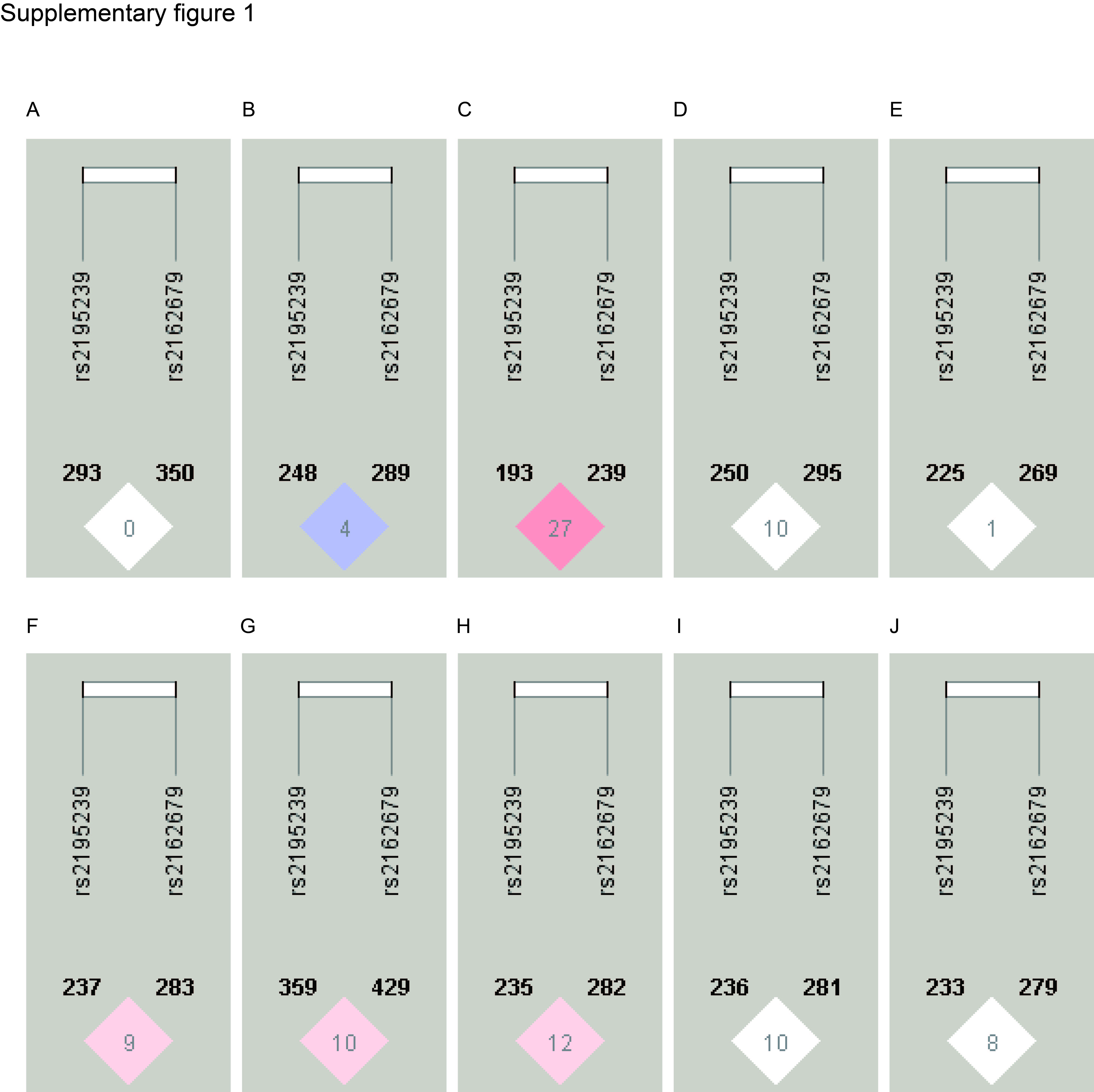

Supplement: Supplementary file 1 — Table S1. Quality score assessment. Table S2. Sensitivity analyses for rs2195239 and rs2162679 polymorphisms and cancer risk. Table S3. MAFs of rs2195239 (genomic position: chromosome12: 102462924) and rs2162679 (genomic position: Chromosome12: 102477481) and polymorphisms in the populations from the 1000 Genomes Project Phase 3. Table S4. Meta-analysis of the association between rs2195239 polymorphism and cancer risk, omitting the study of Patel or Birmann. Table S5. The OMIM numbers for important genes and pathogenic conditions in this study. Figure S1. Linkage disequilibrium analyses for IGF1 rs2195239 and rs2162679 polymorphisms in populations from the 1000 Genomes Project Phase 3. (ZIP 1155 kb) [file 12881_2019_749_MOESM1_ESM.zip › Supplementary Figure 1R4.jpg]
